# Prognostic value of platelet-to-white blood cell ratio in critically ill patients with non-traumatic subarachnoid hemorrhage: a machine learning-driven study of the public database and the hospital information system

**DOI:** 10.3389/fneur.2026.1864288

**Published:** 2026-09-30

**Authors:** Liang Liu, Zhilei Huang, Binbin Gui, Lin Xu, Yansong Liu, Yaolou Wang, Wei Zhang, Haowei Luo, Xiangtong Zhang

**Affiliations:** 1 Department of Neurosurgery, The First Affiliated Hospital of Harbin Medical University, Harbin, Heilongjiang, China; 2 Department of Orthopedics, The First Affiliated Hospital of Harbin Medical University, Harbin, Heilongjiang, China; 3 Department of General Surgery, The First Affiliated Hospital of Harbin Medical University, Harbin, Heilongjiang, China

**Keywords:** platelet-to-white blood cell ratio, non-traumatic subarachnoid hemorrhage, MIMIC-IV, eICU-CRD, hospital information system, machine learning

## Abstract

**Background:**

Assessment of prognosis is crucial for managing non-traumatic subarachnoid hemorrhage (NSAH) patients. The study aimed to explore the association between platelet-to-white blood cell ratio (PWR) and all-cause mortality in this high-risk population and to develop machine learning models to improve prognostic accuracy.

**Methods:**

Using data from the Medical Information Mart for Intensive Care (MIMIC-IV). The observational endpoints were 30-day and 365-day mortality. Patients were categorized into four groups based on the quartiles of PWR. Kaplan–Meier curves, multivariate Cox regression analyses, restricted cubic splines (RCS), and subgroup analyses were applied. We also developed machine learning models that include PWR and clinical features selected with the Boruta and LASSO algorithms to predict 30-day mortality. Model performance was evaluated by the receiver operating characteristic (ROC) curve and the area under the curve (AUC). Shapley Additive exPlanations (SHAP) method was utilized to provide model interpretability. External validations in the eICU-Collaborative Research Database (eICU-CRD) and the Hospital Information System (HIS) of the First Affiliated Hospital of Harbin Medical University were used to assess generalizability.

**Results:**

A total of 1,176 patients with critically ill NSAH were enrolled from MIMIC-IV, predominantly male (58.8%), with a median age of 62 years. In multivariable Cox models, higher PWR quartiles were independently associated with lower risk of 30-day (HR = 0.55, 95% CI 0.35, 0.86, *p* = 0.009) and 365-day (HR = 0.53, 95% CI 0.37, 0.77, *p* = 0.001) mortality after fully adjusting. RCS suggested a linear association. No significant interaction was observed by subgroup analysis across all subgroups. The top-performing machine learning model attained an AUC of 0.868 during internal validation (MIMIC-IV). External validations yielded AUC values of 0.874 (eICU-CRD) and 0.763 (HIS). SHAP analysis revealed a correlation between decreased PWR and an elevated predicted risk of mortality.

**Conclusion:**

PWR is an independent predictor of short- and long-term mortality in ICU patients with NSAH. Machine learning models incorporating PWR significantly improve risk stratification, thereby providing precious support for the early identification and personalized management of patients in the ICU.

## 1 Introduction

Non-traumatic subarachnoid hemorrhage (NSAH) is the third most common type of stroke and is frequently related to aneurysmal rupture (1). The principal clinical manifestations of NSAH are severe headache and nausea with vomiting, both resulting from raised intracranial pressure. Although the global incidence of NSAH is declining (2), early mortality remains high. About a quarter of NSAH patients die before hospital admission. Survivors often develop neurological deficits that reduce quality of life and impose a substantial socioeconomic burden (3). Currently, emergent therapeutic interventions aim to reduce rebleeding risk by managing hypertension and correcting coagulopathies. Prompt aneurysm treatment with coil embolisation or clipping is vital. Early brain injury (EBI) within the first 72 h after NSAH significantly impacts patient prognosis. Uncontrolled inflammation and coagulation issues are key factors in EBI pathophysiology, according to previous studies (4, 5).

Complete blood count (CBC) indices and their ratios, such as neutrophil-to-lymphocyte ratio (NLR) and platelet-to-lymphocyte ratio (PLR), are frequently linked to negative clinical outcomes in various acutely ill cohorts (6–8). The platelet-to-white blood cell ratio (PWR), a biomarker calculated as the absolute platelet (PLT) count divided by the absolute white blood cell (WBC) count from peripheral blood, has recently attracted growing interest. PWR reflects the balance between inflammation and coagulation and indicates the overall systemic inflammatory state and its trajectory of recovery. PWR has been validated as an independent clinical prognostic predictor for various diseases, for instance, atherosclerotic cardiovascular disease (9), pyogenic liver abscesses (10), intracerebral hemorrhage (11), and traumatic acute subdural hematoma (12). However, its predictive value for NSAH patients in the ICU remains unclear.

The increasing access to comprehensive electronic health records and advancements in artificial intelligence (AI) have opened up possibilities for utilizing machine learning in predicting risks in critical illness. Machine learning methods exhibit enhanced capacity in analyzing complex clinical data and detecting nonlinear correlations that traditional statistical approaches often miss, thus surpassing inherent methodological constraints (13). This research aims to develop and validate a machine learning model incorporating PWR for 30-day all-cause mortality prediction, customized for NSAH patients.

## 2 Methods

### 2.1 Data source

This retrospective study extracted data of patients from the three sources. Firstly, data were obtained from the Medical Information Mart for Intensive Care IV database (MIMIC-IV v3.1). This publicly available database includes data from over 94,458 intensive care unit admissions at Beth Israel Deaconess Medical Center in Boston, Massachusetts, spanning 2008 to 2019 (14). One author, Yansong Liu, obtained access to the database after completing the Collaborative Institutional Training Initiative (CITI) certification (Record ID: 59888572).

Secondly, one external validation cohort was performed utilizing the eICU-Collaborative Research Database (eICU-CRD v2.0), a multicenter database that encompasses de-identified health data from over 200,000 ICU admissions across 208 hospitals in the United States during the years 2014 and 2015 (15). As all data in MIMIC-IV and eICU-CRD have undergone de-identification, informed consent was waived.

Thirdly, the other external validation cohort was sourced from the Hospital Information System (HIS) of the First Affiliated Hospital of Harbin Medical University in China, covering the period from July 2024 to June 2025. The HIS dataset thoroughly documents a range of clinical parameters recorded during ICU hospitalization. All data underwent stringent de-identification procedures in compliance with ethical standards to safeguard confidentiality and information security. The study protocol was reviewed and approved by the Ethics Committee of the First Affiliated Hospital of Harbin Medical University, with informed consent acquired from either the patients or their legal guardians.

### 2.2 Study population

Based on previous research (16), patient information matching non-traumatic subarachnoid hemorrhage (NSAH) diagnoses according to the International Classification of Diseases (ICD-9 codes 430 or ICD-10 codes beginning with I60) was extracted from the MIMIC-IV database. Exclusion criteria were: (1) age < 18 years; (2) patients who were not the first ICU admission; (3) ICU length of stay < 24 h; and (4) missing PLT or WBC measurements. Ultimately, 1,176 patients were included in the study. All eligible patients from the MIMIC-IV database were randomly split into two cohorts: a training cohort (n = 823) for model development and a testing cohort (*n* = 353) for internal model evaluation. For external validation, following the same criteria as the MIMIC-IV database, 896 and 513 eligible patients with NSAH were identified from the eICU-CRD database and the HIS dataset, respectively. The flow chart of the study is presented in Figure 1.

**Figure 1:**
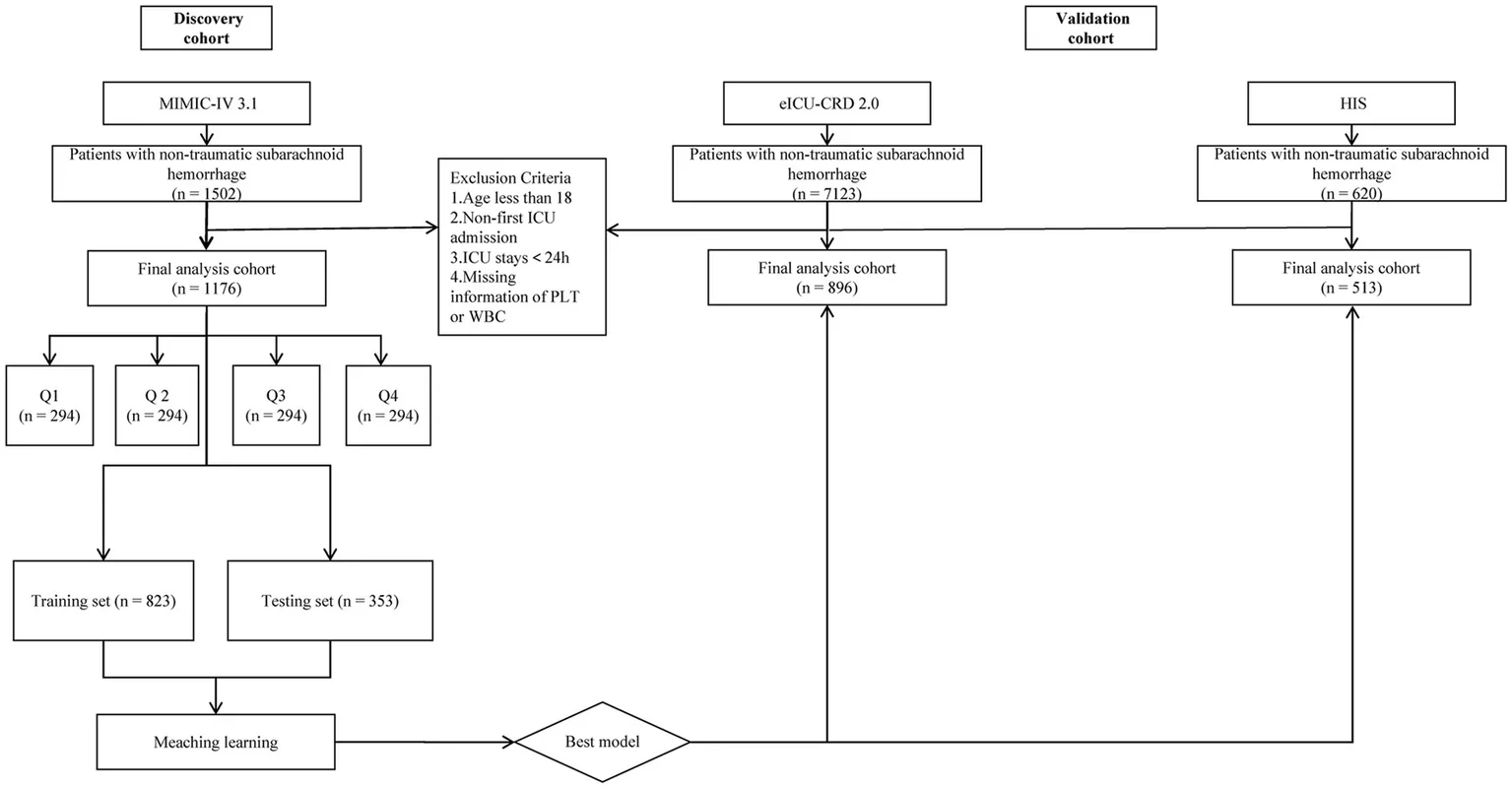
The flowchart of patients in different cohorts and modeling.

### 2.3 Calculation of PWR

PWR was chosen as the primary study variable, and it was calculated as platelets (10^9/L) divided by white blood cells (WBC) (10^9/L) (17). The 25th, 50th, and 75th percentiles were calculated based on the distribution of the PWR at ICU admission. Subsequently, the NSAH patients were categorized into four groups according to the critical values of these quartiles: Q1: < 14.38; Q2: 14.38–19.07; Q3: 19.07–25.22; Q4: ≥ 25.22.

### 2.4 Data collection

Data from the MIMIC-IV and eICU-CRD databases were extracted utilizing Navicate Premium (v17) by executing Structured Query Language (SQL) queries. The extraction of variables was divided into six primary categories: demographic, laboratory results, vital signs, comorbidities, clinical severity scores, and treatments. More details are provided in Supplementary Table S1. The laboratory results, vital signs, and clinical severity scores within the first 24 h after ICU admission were collected. In MIMIC-IV, the primary outcomes were 30-day mortality and 365-day mortality. In the external validation cohorts, the primary outcome was in-hospital mortality. Based on Supplementary Table S2, variables with a missing percentage > 20% were removed, and those ≤ 20% were imputed utilizing multiple imputation with the “mice” package in R software.

### 2.5 Statistical analysis

Continuous variables were described as mean ± standard deviation (SD) for normally distributed variables, median (Interquartile Range, IQR) for non-normally distributed continuous variables, and their differences were analyzed utilizing the t-test or Mann–Whitney U-test. Categorical variables were reported as frequencies and percentages (%) and compared using the Chi-square test or Fisher’s exact test. Kaplan–Meier survival analysis curves were drawn to assess 30-day and 365-day survival across PWR quartiles, and differences between the curves were compared utilizing the log-rank test. Before fitting multivariable models, all variables were assessed for the variance inflation factor (VIF). Variables with a VIF ≥ 5 were considered collinear and were excluded (Supplementary Table S3). Multivariate Cox regression analyses examined the association between PWR and the two outcomes (30-day and 365-day mortality), while in-hospital mortality was evaluated through multivariate logistic regression. The results were reported as hazard ratios (HRs) or odds ratios (ORs) along with 95% confidence intervals (CIs). As for the MIMIC-IV cohort, the adjustments in model 3 were determined by the Venn diagrams together with clinical judgment. Restricted cubic spline (RCS) models were utilized to evaluate potential non-linear relationships between PWR and all-cause mortality. Subgroup analyses were conducted to confirm the reliability of the results. Likelihood ratio tests were employed to investigate interactions between PWR and variables utilized for stratification. *p*-value < 0.05 was considered to be statistically significant. Statistical analysis was carried out using R software (v4.0.2) and SPSS 22.0.

### 2.6 Model development and validation

Prior to model development, data from the MIMIC-IV cohort were partitioned into training (70%) and testing (30%) sets to mitigate overfitting. In the training set, data extracted from the databases underwent processing to address outliers and missing values. Outliers were identified using the IQR method, which defines them as values exceeding 1.5 times the IQR above the third quartile or below the first quartile. Variables with a missing percentage greater than 20% were excluded from the analysis. For the remaining variables, we employed multiple imputation by chained equations to handle missing data. To minimize potential selection bias and model instability, we independently applied both the Boruta and LASSO algorithms for variable screening within the training set (18–20), resulting in two validated sets of significant predictors. To ensure the inclusion of only the most relevant and robust variables in the predictive model while reducing the risks of overfitting or incorporating extraneous predictors, we utilized the intersection of the two predictive feature sets as the final feature set in the model. Model hyperparameters were optimized within the training set. Subsequently, all preprocessing parameters derived from the training set were fixed and applied to the testing set and external validation cohorts. Data from eICU-CRD and HIS served as the external sets. Seven machine learning models were developed, specifically: Logistic Regression (LR), Decision Tree (DT), Random Forest (RF), Extreme Gradient Boosting (XGBoost), Light Gradient Boosting Machine (LightGBM), Support Vector Machine (SVM), and Artificial Neural Network (ANN). We assessed the performance of models using the receiver operating characteristic (ROC) curve and the area under the curve (AUC). Accuracy, precision, sensitivity, specificity, and F1-score were also selected to evaluate the discrimination of the model. Additional evaluations encompassed decision curve analysis (DCA) and calibration curve analysis. The top-performing model from MIMIC IV underwent external validation on the eICU-CRD and HIS cohorts. Lastly, the interpretability of the ultimate prediction model was assessed utilizing the Shapley Additive exPlanations (SHAP) method. The SHAP values were computed exclusively using the testing set.

## 3 Results

### 3.1 Baseline characteristics

A total of 1,176 critically ill patients with NSAH from the MIMIC-IV database were finally enrolled in this study. Patients were divided into four groups by admission PWR: Q1 (PWR < 14.38), Q2 (14.38 ≤ PWR < 19.07), Q3 (19.07 ≤ PWR < 25.22), and Q4 (PWR ≥ 25.22). The median age of the included patients was 62 years, with an interquartile range of 46 to 77 years, and males accounted for 58.8 percent. The 30-day mortality and 365-day mortality after admission were 21.3% (n = 250) and 29.3% (n = 345), respectively. In comparison with Q4, the Q1 group had low PWR values and PLT; nevertheless, their WBC, potassium, AG, creatinine, BUN, RR, HR, urine output, and clinical severity scores (APSIII, OASIS, SOFA, SAPSII, SIRS) were higher. Additionally, the 30-day mortality was lower in groups with higher PWR values (Q4:9.9% vs. Q1:37.1%). The same pattern held for 365-day mortality (Q4:15.3% vs. Q1:49.3%). These trends were statistically significant (all *p* < 0.001). Severe comorbidities (CHF, malignant cancer, AKI, sepsis) were more common in Q1 patients, who also had higher CCI. In terms of clinical treatments, dopamine, epinephrine, norepinephrine, mannitol, and furosemide were utilized more often in Q1. Ventilator and CRRT were also more common in patients with low PWR. More details are demonstrated in Table 1.

**Table 1:** Baseline characteristics according to PWR quartiles.

Characteristic	ALL	Q1	Q2	Q3	Q4	*p*-value
	(*n* = 1,176)	PWR < 14.38 (*n* = 294)	14.38 ≤ PWR < 19.07 (*n* = 294)	19.07 ≤ PWR < 25.22 (*n* = 294)	PWR ≥ 25.22 (*n* = 294)	
Demographic						
Age, (years)	61.61 ± 15.35	61.73 ± 15.14	62.04 ± 16.09	60.95 ± 15.71	61.74 ± 14.46	0.849
Gender, *n* (%)						**<0.001**
Female	485 (41.2)	150 (51.0)	119 (40.5)	115 (39.1)	101 (34.4)	
Male	691 (58.8)	144 (49.0)	175 (59.5)	179 (60.9)	193 (65.6)	
Race, *n* (%)						**<0.001**
Other	660 (56.1)	138 (46.9)	150 (51.0)	164 (55.8)	208 (70.7)	
White	516 (43.9)	156 (53.1)	144 (49.0)	130 (44.2)	86 (29.3)	
Laboratory results						
WBC, (10^9/L)	12.10 ± 5.47	16.60 ± 7.11	12.73 ± 3.78	10.62 ± 2.82	8.45 ± 3.33	**<0.001**
PLT, (10^9/L)	220.21 ± 88.40	164.02 ± 77.20	212.54 ± 61.79	231.17 ± 60.48	273.12 ± 108.37	**<0.001**
APTT, (s)	32.92 ± 19.44	35.01 ± 21.64	33.73 ± 22.96	30.83 ± 15.25	32.12 ± 16.64	0.05
Na+, (mmol/L)	139.28 ± 4.12	139.01 ± 4.88	139.70 ± 3.99	139.29 ± 3.90	139.13 ± 3.60	0.195
K+, (mmol/L)	3.94 ± 0.62	4.04 ± 0.86	3.86 ± 0.55	3.96 ± 0.54	3.89 ± 0.43	**0.002**
Cl−, (mmol/L)	104.74 ± 5.01	104.41 ± 5.89	104.90 ± 5.20	104.94 ± 4.43	104.72 ± 4.39	0.568
AG	14.28 ± 3.58	15.65 ± 4.25	14.45 ± 3.48	13.78 ± 3.07	13.23 ± 2.93	**<0.001**
Creatinine, (mg/dL)	0.96 ± 0.88	1.19 ± 0.93	0.99 ± 1.21	0.86 ± 0.66	0.81 ± 0.53	**<0.001**
BUN, (mmol/L)	16.75 ± 12.71	22.38 ± 19.30	16.16 ± 10.44	14.21 ± 7.86	14.24 ± 7.70	**<0.001**
Vital signs						
Temperature, (°C)	36.81 ± 0.78	36.75 ± 1.01	36.86 ± 0.69	36.83 ± 0.66	36.81 ± 0.70	0.424
RR, (times/min)	18.25 ± 5.19	19.47 ± 6.17	18.19 ± 5.14	17.90 ± 4.41	17.46 ± 4.67	**<0.001**
SBP, (mmHg)	131.81 ± 22.30	130.75 ± 24.25	132.43 ± 23.09	130.49 ± 21.49	133.55 ± 20.14	0.295
DBP, (mmHg)	71.99 ± 16.41	71.88 ± 18.32	70.96 ± 16.52	71.67 ± 14.88	73.43 ± 15.69	0.316
HR, (times/min)	81.98 ± 17.59	85.60 ± 19.75	83.52 ± 17.11	79.50 ± 16.37	79.31 ± 16.15	**<0.001**
SpO2, (%)	97.46 ± 3.07	97.48 ± 3.33	97.73 ± 2.87	97.19 ± 3.39	97.45 ± 2.60	0.199
Urine output, (mL)	24239.95 ± 27229.88	26621.93 ± 31668.68	26270.80 ± 28112.46	23337.80 ± 23801.49	20729.27 ± 24304.64	**0.028**
Comorbidities, *n* (%)						
Hypertension						0.08
No	590 (50.2)	166 (56.5)	146 (49.7)	137 (46.6)	141 (48.0)	
Yes	586 (49.8)	128 (43.5)	148 (50.3)	157 (53.4)	153 (52.0)	
Diabetes						0.141
No	991 (84.3)	238 (81.0)	244 (83.0)	252 (85.7)	257 (87.4)	
Yes	185 (15.7)	56 (19.0)	50 (17.0)	42 (14.3)	37 (12.6)	
Hyperlipidemia						0.289
No	841 (71.5)	208 (70.7)	218 (74.1)	199 (67.7)	216 (73.5)	
Yes	335 (28.5)	86 (29.3)	76 (25.9)	95 (32.3)	78 (26.5)	
CHF						**0.001**
No	1,066 (90.6)	256 (87.1)	257 (87.4)	274 (93.2)	279 (94.9)	
Yes	110 (9.4)	38 (12.9)	37 (12.6)	20 (6.8)	15 (5.1)	
CPD						0.206
No	1,016 (86.4)	247 (84.0)	264 (89.8)	251 (85.4)	254 (86.4)	
Yes	160 (13.6)	47 (16.0)	30 (10.2)	43 (14.6)	40 (13.6)	
Malignant cancer						**0.002**
No	1,121 (95.3)	269 (91.5)	285 (96.9)	287 (97.6)	280 (95.2)	
Yes	55 (4.7)	25 (8.5)	9 (3.1)	7 (2.4)	14 (4.8)	
AKI						**<0.001**
No	305 (25.9)	49 (16.7)	68 (23.1)	88 (29.9)	100 (34.0)	
Yes	871 (74.1)	245 (83.3)	226 (76.9)	206 (70.1)	194 (66.0)	
Sepsis						**<0.001**
No	616 (52.4)	101 (34.4)	139 (47.3)	185 (62.9)	191 (65.0)	
Yes	560 (47.6)	193 (65.6)	155 (52.7)	109 (37.1)	103 (35.0)	
CCI	4.24 ± 2.56	4.78 ± 2.62	4.19 ± 2.63	3.94 ± 2.52	4.04 ± 2.40	**<0.001**
Clinical severity scores						
APSIII	38.28 ± 20.18	49.23 ± 24.77	37.84 ± 18.31	33.20 ± 16.50	32.85 ± 15.35	**<0.001**
OASIS	30.74 ± 8.41	33.71 ± 8.64	31.87 ± 8.36	28.62 ± 7.91	28.74 ± 7.62	**<0.001**
SOFA	2.67 ± 2.43	4.35 ± 3.25	2.54 ± 1.97	1.96 ± 1.60	1.84 ± 1.60	**<0.001**
GCS	14.29 ± 1.27	14.19 ± 1.40	14.22 ± 1.34	14.42 ± 1.03	14.33 ± 1.28	0.11
SAPSII	33.03 ± 13.38	39.78 ± 15.57	33.27 ± 12.83	29.53 ± 10.78	29.55 ± 11.17	**<0.001**
SIRS	2.44 ± 1.03	2.95 ± 0.90	2.62 ± 0.97	2.24 ± 0.95	1.93 ± 0.99	**<0.001**
Treatments, *n* (%)						
Dobutamine						0.077
No	1,166 (99.1)	289 (98.3)	290 (98.6)	294 (100.0)	293 (99.7)	
Yes	10 (0.9)	5 (1.7)	4 (1.4)	0 (0.0)	1 (0.3)	
Dopamine						**0.002**
No	1,161 (98.7)	284 (96.6)	291 (99.0)	293 (99.7)	293 (99.7)	
Yes	15 (1.3)	10 (3.4)	3 (1.0)	1 (0.3)	1 (0.3)	
Epinephrine						**0.001**
No	1,145 (97.4)	277 (94.2)	287 (97.6)	290 (98.6)	291 (99.0)	
Yes	31 (2.6)	17 (5.8)	7 (2.4)	4 (1.4)	3 (1.0)	
Norepinephrine						**<0.001**
No	947 (80.5)	196 (66.7)	238 (81.0)	253 (86.1)	260 (88.4)	
Yes	229 (19.5)	98 (33.3)	56 (19.0)	41 (13.9)	34 (11.6)	
Vasopressin						**<0.001**
No	1,071 (91.1)	243 (82.7)	268 (91.2)	283 (96.3)	277 (94.2)	
Yes	105 (8.9)	51 (17.3)	26 (8.8)	11 (3.7)	17 (5.8)	
Mannitol						**<0.001**
No	1,015 (86.3)	229 (77.9)	251 (85.4)	264 (89.8)	271 (92.2)	
Yes	161 (13.7)	65 (22.1)	43 (14.6)	30 (10.2)	23 (7.8)	
Furosemide						**<0.001**
No	852 (72.4)	170 (57.8)	201 (68.4)	242 (82.3)	239 (81.3)	
Yes	324 (27.6)	124 (42.2)	93 (31.6)	52 (17.7)	55 (18.7)	
Clipping of aneurysm						0.492
No	1,154 (98.1)	289 (98.3)	288 (98.0)	286 (97.3)	291 (99.0)	
Yes	22 (1.9)	5 (1.7)	6 (2.0)	8 (2.7)	3 (1.0)	
Ventilator						**<0.001**
No	242 (20.6)	32 (10.9)	43 (14.6)	76 (25.9)	91 (31.0)	
Yes	934 (79.4)	262 (89.1)	251 (85.4)	218 (74.1)	203 (69.0)	
CRRT						**<0.001**
No	1,143 (97.2)	272 (92.5)	287 (97.6)	292 (99.3)	292 (99.3)	
Yes	33 (2.8)	22 (7.5)	7 (2.4)	2 (0.7)	2 (0.7)	
Clinical outcome						
30-day mortality	250 (21.3)	109 (37.1)	62 (21.1)	50 (17.0)	29 (9.9)	**<0.001**
365-day mortality	345 (29.3)	145 (49.3)	85 (28.9)	70 (23.8)	45 (15.3)	**<0.001**

PWR, platelet-to-white cell ratio; WBC, white blood cell; PLT, platelet; APTT, Activated Partial Thromboplastin Time; Na+, sodium; K+, potassium; Cl−:chloride; AG, anion gap; BUN, blood urea nitrogen; RR, respiratory rate; SBP, systolic blood pressure; DBP, Diastolic blood pressure; HR, heart rate; Spo2, peripheral oxygen saturation; CHF, congestive heart failure; CPD, chronic pulmonary disease; AKI, acute kidney injury; CCI, Charlson Comorbidity Index; APSIII, Acute Physiology Score III; OASIS, Oxford Acute Severity of ILLness Score; SOFA, the Sequential Organ-Failure Assessment; GCS, the Glasgow Coma Scale; SAPSII, Simplified Acute Physiology Score II; SIRS, Systemic Inflammatory Response Syndrome; CRRT, Continuous Renal Replacement Therapy. The bold values in Table 1 indicate a p-value <0.05.

### 3.2 Kaplan–Meier curve

Figure 2 shows the Kaplan–Meier survival curve analyses for 30-day mortality and 365-day mortality across PWR quartiles. Patients with higher PWR at 30 days after ICU admission had a higher survival probability compared with those in the lowest quartile (log-rank, *p* < 0.0001). The same pattern persisted at 365 days (Q4 > Q3 > Q2 > Q1).

**Figure 2:**
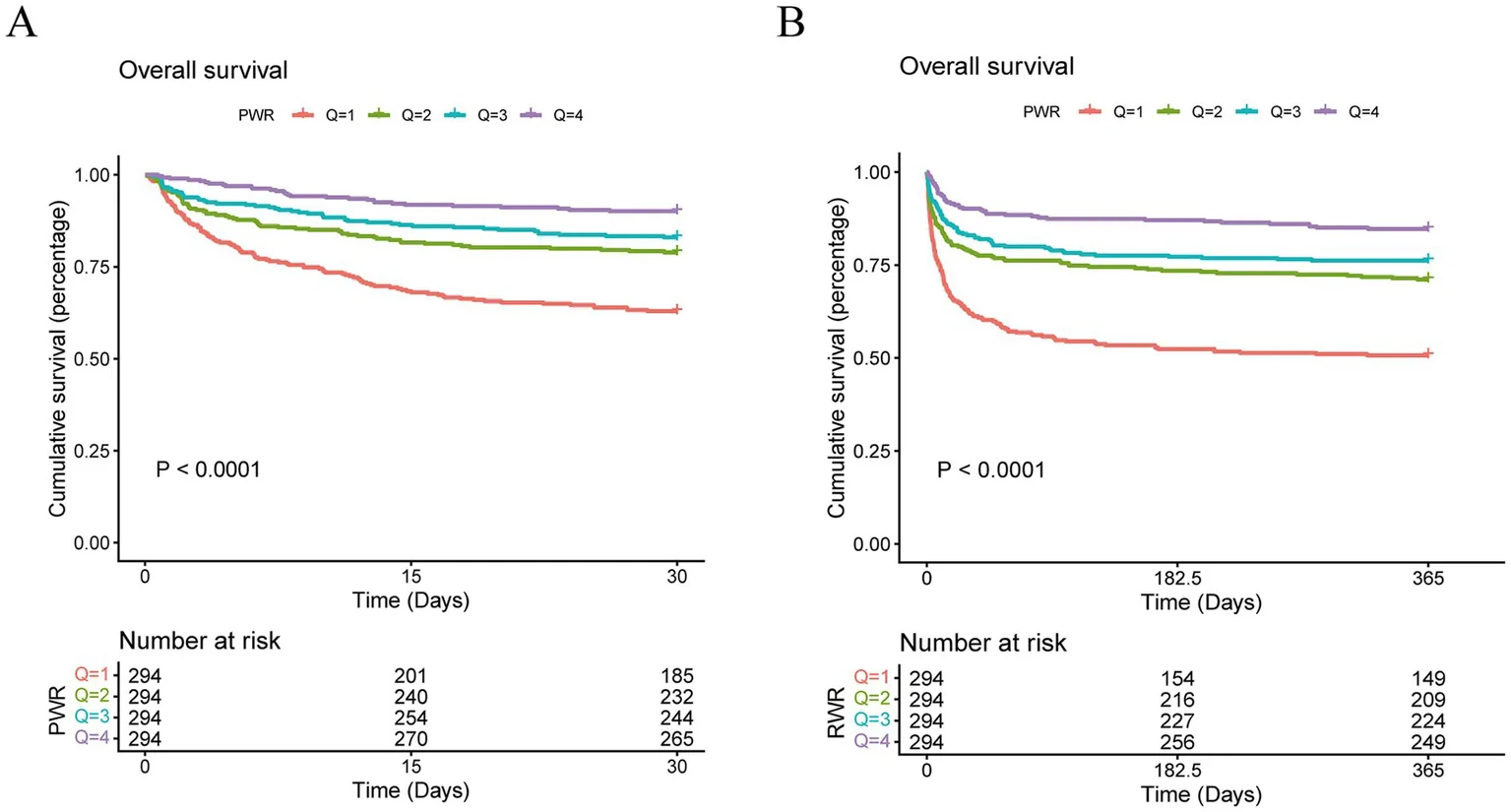
Kaplan–Meier survival analysis curves for all-cause mortality by PWR quartiles. **(A)** 30-day mortality; **(B)** 365-day mortality.

### 3.3 Multivariate cox regression (MIMIC-IV) and logistic regression models (eICU-CRD and HIS)

To investigate the association between PWR and all-cause mortality in patients with NSAH, three multivariate Cox regression analyses were applied. As displayed in Table 2, model 1 was unadjusted, model 2 was adjusted for age, gender, race, and model 3 was adjusted for age, gender, race, BUN, creatinine, AG, hypertension, diabetes, RR, temperature, CCI, OASIS, SAPSII, SIRS, norepinephrine, vasopressin, and mannitol. When PWR was analyzed as a categorical variable, the fully adjusted model 3 revealed that the highest PWR quartiles were associated with lower risk of 30-day (Q4 vs. Q1: HR = 0.55, 95% CI 0.35, 0.86, *p* = 0.009, P for trend = 0.018) and 365-day (Q4 vs. Q1: HR = 0.53, 95% CI 0.37, 0.77, *p* = 0.001, P for trend = 0.002) all-cause mortality. Similarly, as a continuous variable, higher PWR remained associated with lower 30-day (HR = 0.98, 95% CI 0.97, 0.99, *p* = 0.001) and 365-day (HR = 0.95, 95% CI 0.97, 0.99, *p* < 0.001) all-cause mortality.

**Table 2:** Relationship between PWR and mortality in critically ill patients with NSAH (discovery cohort and validation cohort).

Categories	Model 1	*p*-value	P for trend	Model 2	*p*-value	P for trend	Model 3	*p*-value	P for trend
	HR/OR (95% CI)			HR/OR (95% CI)			HR/OR (95% CI)		
MIMIC-IV (discovery cohort)									
30-day mortality									
PWR	0.93[0.92, 0.95]	<0.001		0.94[0.92, 0.95]	<0.001		0.98[0.96, 0.99]	0.005	
PWR quartiles			<0.001			<0.001			0.018
Q1	Ref			Ref			Ref		
Q2	0.52 [0.38, 0.71]	<0.001		0.54 [0.40, 0.74]	<0.001		0.90 [0.64, 1.26]	0.523	
Q3	0.40 [0.29, 0.56]	<0.001		0.43 [0.31, 0.60]	<0.001		1.02 [0.70, 1.47]	0.929	
Q4	0.22 [0.15, 0.33]	<0.001		0.25 [0.16, 0.38]	<0.001		0.55 [0.35, 0.86]	0.009	
365-day mortality									
PWR	0.94[0.92, 0.95]	<0.001		0.94[0.93, 0.95]	<0.001		0.98 [0.97, 0.99]	0.001	
PWR quartiles			<0.001			<0.001			0.002
Q1	Ref			Ref			Ref		
Q2	0.52 [0.39, 0.66]	<0.001		0.52 [0.40, 0.68]	<0.001		0.80 [0.60, 1.07]	0.129	
Q3	0.40 [0.30, 0.53]	<0.001		0.42 [0.32, 0.56]	<0.001		0.91 [0.67, 1.24]	0.554	
Q4	0.24 [0.17, 0.34]	<0.001		0.26 [0.18, 0.36]	<0.001		0.53 [0.37, 0.77]	0.001	
eICU-CRD (validation cohort)									
In-hospital mortality									
PWR	0.93 [0.90, 0.95]	<0.001		0.93 [0.90, 0.95]	<0.001		0.95 [0.93, 0.98]	0.001	
PWR quartiles			<0.001			<0.001			0.04
Q1	Ref			Ref			Ref		
Q2	0.51 [0.32, 0.80]	0.004		0.51 [0.31, 0.81]	0.005		0.67 [0.39, 1.13]	0.135	
Q3	0.39 [0.24, 0.63]	<0.001		0.39 [0.24, 0.64]	<0.001		0.63 [0.36, 1.10]	0.107	
Q4	0.31 [0.18, 0.52]	<0.001		0.32 [0.18, 0.53]	<0.001		0.53 [0.29, 0.95]	0.036	
HIS (validation cohort)									
In-hospital mortality									
PWR	0.87 [0.81, 0.94]	<0.001		0.86 [0.79, 0.93]	<0.001		0.87 [0.80, 0.94]	0.001	
PWR quartiles			0.013			0.008			0.021
Q1	Ref			Ref			Ref		
Q2	0.72 [0.27, 1.83]	0.487		0.66 [0.25, 1.73]	0.405		0.76 [0.27, 2.09]	0.600	
Q3	0.53 [0.18, 1.43]	0.222		0.53 [0.17, 1.46]	0.229		0.66 [0.21, 1.93]	0.462	
Q4	0.17 [0.03, 0.65]	0.023		0.14 [0.02, 0.56]	0.014		0.16 [0.02, 0.67]	0.025	

Model 1, Unadjusted.

Model 2, adjusted for age, gender, and race.

Model 3 (MIMIC-IV cohort), adjusted for age, gender, race, BUN, blood urea nitrogen, creatinine; AG, anion gap, hypertension, diabetes; RR, respiratory rate, temperature; CCI, Charlson Comorbidity Index; OASIS, Oxford Acute Severity of ILLness Score; SAPSII, Simplified Acute Physiology Score II; SIRS, Systemic Inflammatory Response Syndrome, norepinephrine, vasopressin, and mannitol.

Model 3 (eICU-CRD cohort): adjusted for age, gender, race, BUN, creatinine, AG, hypertension, diabetes, RR, respiratory rate, temperature; SOFA, the Sequential Organ-Failure Assessment, vasopressin, mannitol, and furosemide.

Model 3 (HIS cohort): adjusted for age, gender, race, BUN, blood urea nitrogen, creatinine; AG, anion gap, hypertension, diabetes; RR, respiratory rate, temperature; GCS, the Glasgow Coma Scale; SIRS, Systemic Inflammatory Response Syndrome, norepinephrine, vasopressin, mannitol, furosemide, and clipping of aneurysm.

The two external validation cohorts were from the eICU-CRD (America) and the HIS (China). Similarly, we utilized three multivariate logistic regression models to investigate the association between PWR and in-hospital mortality in patients with NSAH. In the eICU-CRD database, after fully adjusting for age, gender, race, BUN, creatinine, AG, hypertension, diabetes, RR, temperature, SOFA, vasopressin, mannitol, and furosemide, patients in the highest PWR group had a significantly decreased risk of in-hospital mortality (Q4 vs. Q1: OR = 0.53, 95% CI 0.29, 0.59, *p* = 0.036, P for trend = 0.04). Additionally, higher PWR as a continuous variable was consistently associated with lower in-hospital mortality (OR = 0.95, 95% CI 0.93, 0.98, *p* = 0.001). In the other validation cohort in China, after fully adjusting for age, gender, race, BUN, creatinine, AG, hypertension, diabetes, RR, temperature, GCS, SIRS, norepinephrine, vasopressin, mannitol, furosemide, and clipping of aneurysm, the HIS showed the same association (Q4 vs. Q1: OR = 0.16, 95% CI 0.06, 0.67, *p* = 0.025, P for trend = 0.021). Detailed data are presented in Table 2. Consequently, our findings highlight the significant predictive benefits of the PWR.

### 3.4 Restricted cubic spline

As demonstrated in the restricted cubic spline model (Figure 3), a significant linear association was observed between NSAH and the risk of 30-day (P for overall = 0.037; P for nonlinear = 0.944) and 365-day (P for overall = 0.001; P for nonlinear = 0.321) all-cause mortality after ICU admission, while the nonlinear relationship was not significant.

**Figure 3:**
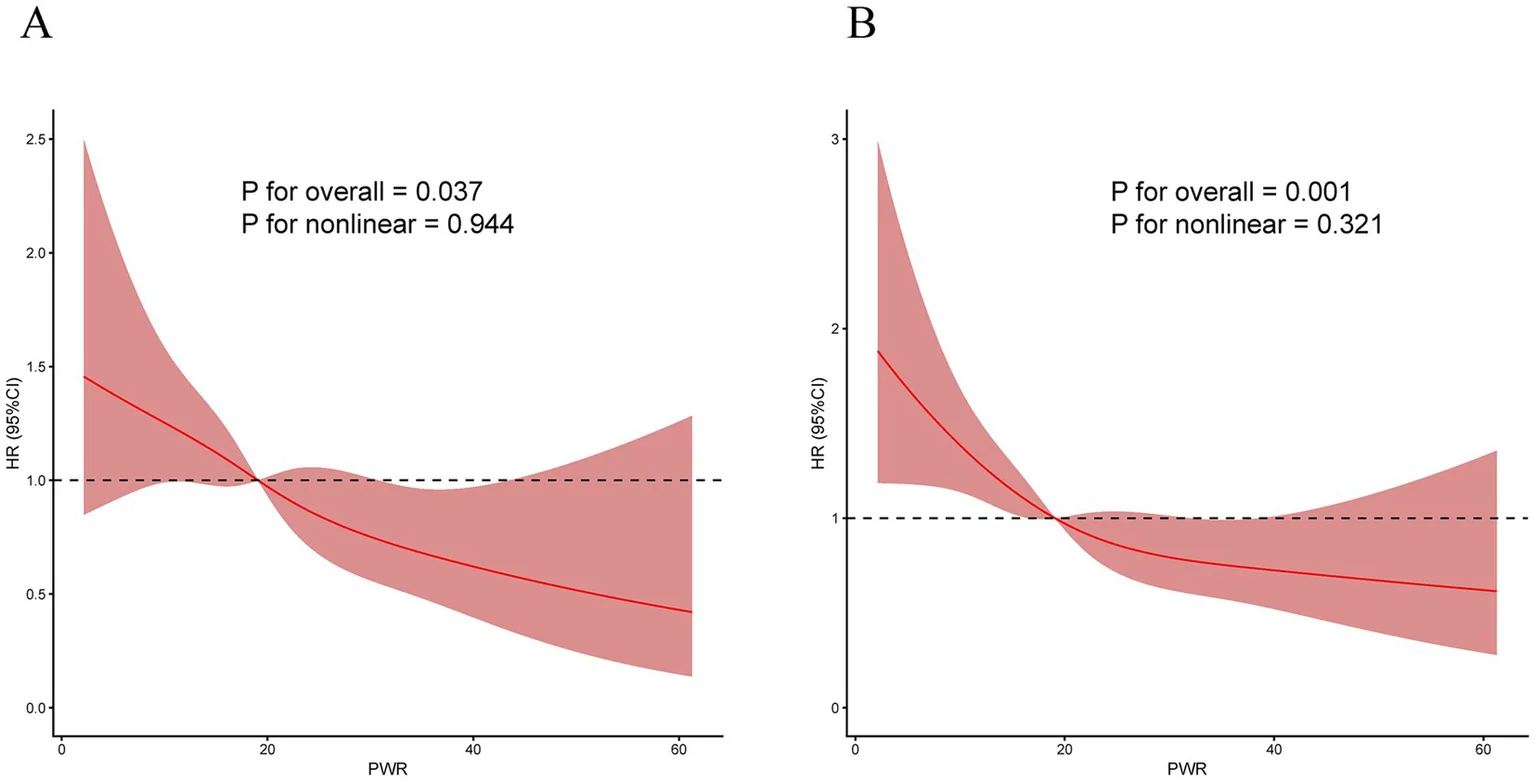
Association between PWR and 30-day and 365-day mortality in the restricted cubic spline plots. **(A)** 30-day mortality; **(B)** 365-day mortality.

### 3.5 Subgroup analysis

To validate the robustness and consistency of the study results, subgroup analyses were undertaken to appraise the connection of PWR with 30-day and 365-day mortality (Figure 4). Overall, the link between PWR and 30-day mortality remains robust across subgroups, with greater PWR values linked to lower mortality rates. The same pattern can be observed at 365 days. When stratified analyses were performed for age, gender, race, hypertension, and diabetes, the forest plots showed no significant interaction between PWR and most subgroups (*p* > 0.05). However, a slight interaction between PWR and age subgroups was noted at 365 days (P for interaction = 0.05).

**Figure 4:**
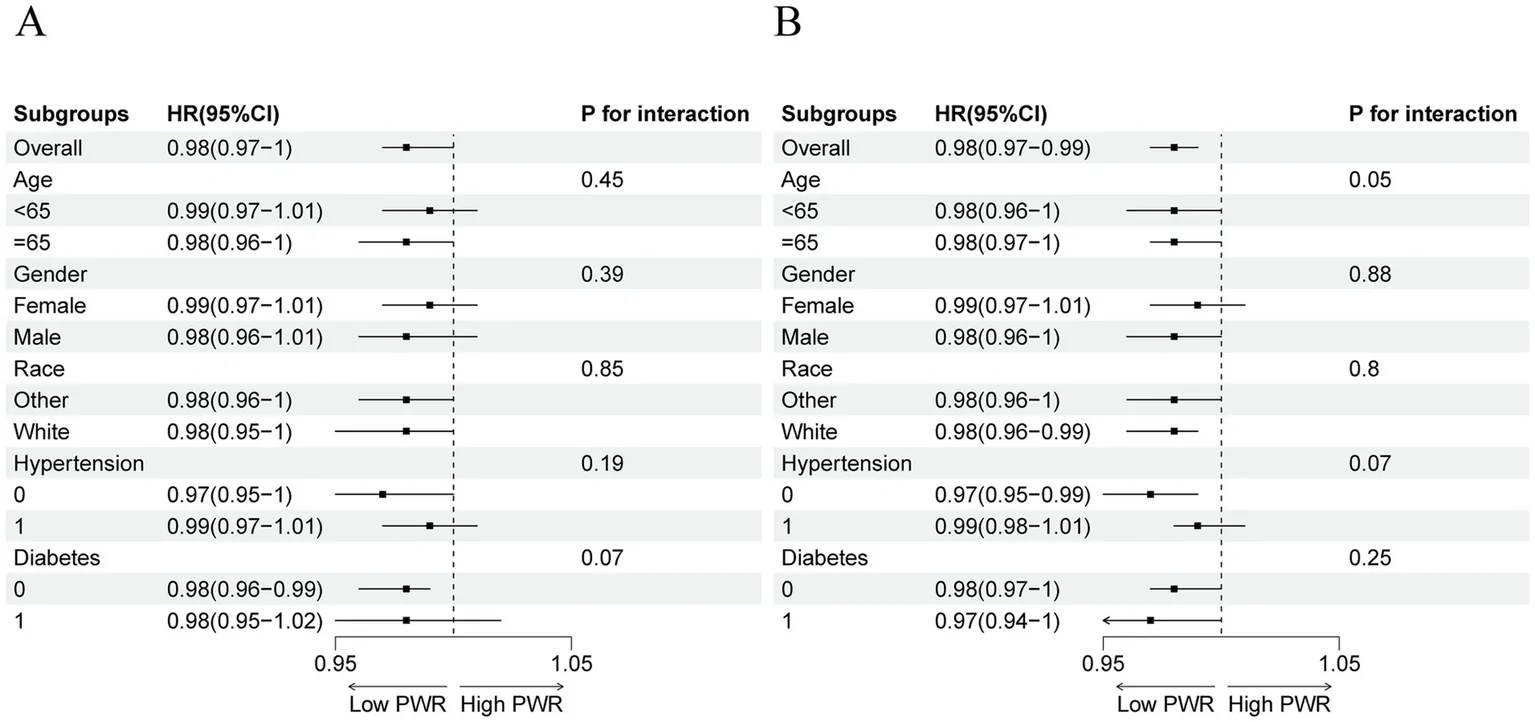
Subgroup analyses of the association of PWR with all-cause mortality. **(A)** 30-day mortality; **(B)** 365-day mortality.

### 3.6 Feature selection

In this study, two feature selection methods were utilized to identify variables with significant predictive value for the 30-day mortality risk in NSAH patients. After the calculation, the top 25 features were obtained by the Boruta algorithm (Figure 5A). Moreover, the path diagrams and crossover curves of the LASSO regression showed the included features (Figures 5B,C). The combined results of both methods are visualized in the Venn plots (Figure 5D). Ultimately, fifteen features, AG, creatinine, BUN, PWR, temperature, RR, urine output, glucose, CCI, OASIS, SAPSIII, SIRS, norepinephrine, vasopressin, and mannitol, were selected for inclusion in the modelling.

**Figure 5:**
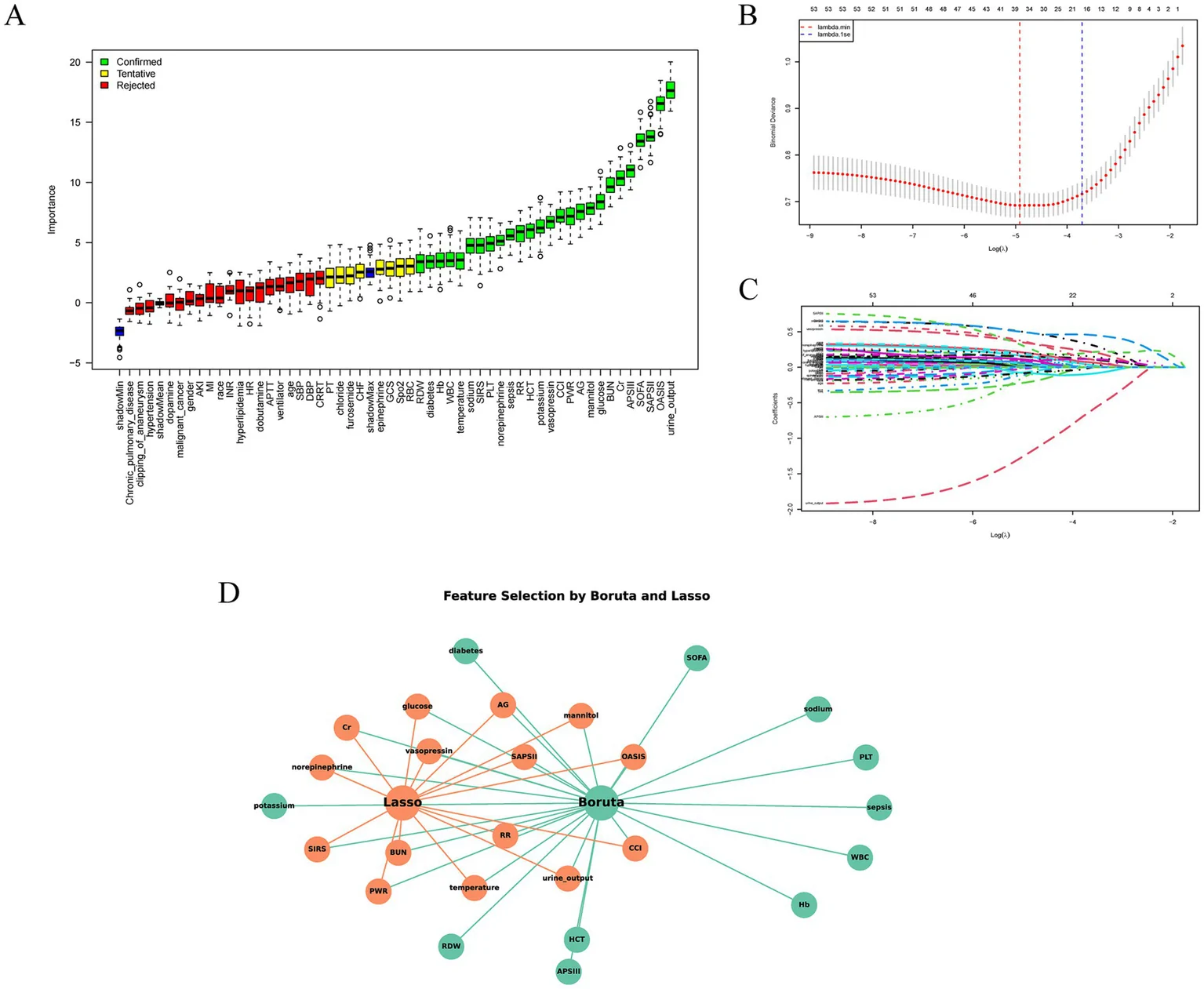
PWR and 30-day mortality feature selection chart. **(A)** Feature selection based on the Boruta algorithm; **(B,C)** Path diagrams and cross-validation plots of LASSO regression analysis results; **(D)** Venn diagrams for feature selection. The orange section shows the results of the LASSO regression analysis, and the green section shows the results of the Boruta algorithm. The final features selected are AG, creatinine, BUN, PWR, temperature, RR, urine output, glucose, CCI, OASIS, SAPSII, SIRS, norepinephrine, vasopressin, and mannitol.

### 3.7 Model development and internal validation

Seven machine learning algorithms were developed for predicting 30-day mortality risk in NSAH patients: LR, DT, RF, XGBoost, LightGBM, SVM, and ANN. All models performed well on the testing set (Figure 6). Concrete details of the training set are shown in Supplementary Figure S1. The ANN model exhibited the most robust and stable discriminative performance, with an AUC of 0.868 and precision of 0.596, sensitivity of 0.453, specificity of 0.917, overall accuracy of 0.819, balanced accuracy of 0.685, and the highest F1 score of 0.515, as depicted in Figure 6A and Supplementary Table S4. DCA in Figure 6B illustrated that ANN provided greater net benefit than the treat-all and treat-none strategies over a wide range of threshold probabilities, excelling particularly between thresholds of 0.1 to 0.6. Furthermore, calibration curves (Figure 6C) showed that LR, XGBoost, and ANN had the best agreement between predicted and observed outcomes. Collectively, ANN was chosen as the ultimate best model for further interpretation and external validation.

**Figure 6:**
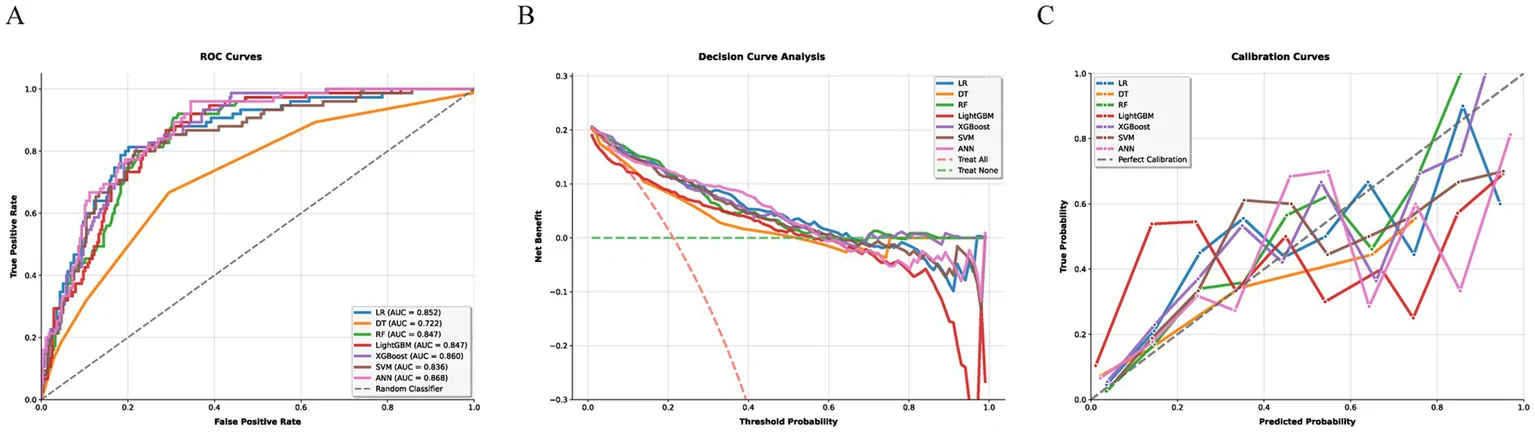
Seven machine learning models in internal validation (MIMIC-IV). **(A)** ROC curves; **(B)** Decision curve analysis; **(C)** Calibration curves.

### 3.8 External validation of best model

To further validate the generalizability of our constructed model, the best model ANN was externally validated using the eICU-CRD cohort and the HIS cohort. It maintained strong discrimination for predicting in-hospital mortality, with AUCs of 0.874 and 0.763, respectively (Figures 7A,D). DCA (Figures 7B,E) demonstrated higher net benefit than both treat-all and treat-none strategies across clinically relevant thresholds. Additionally, in the two transnational external validation sets, the calibration curves (Figures 7C,F) demonstrated good agreement between predicted and observed outcomes. Overall, these findings support its clinical applicability and strong generalizability across diverse populations.

**Figure 7:**
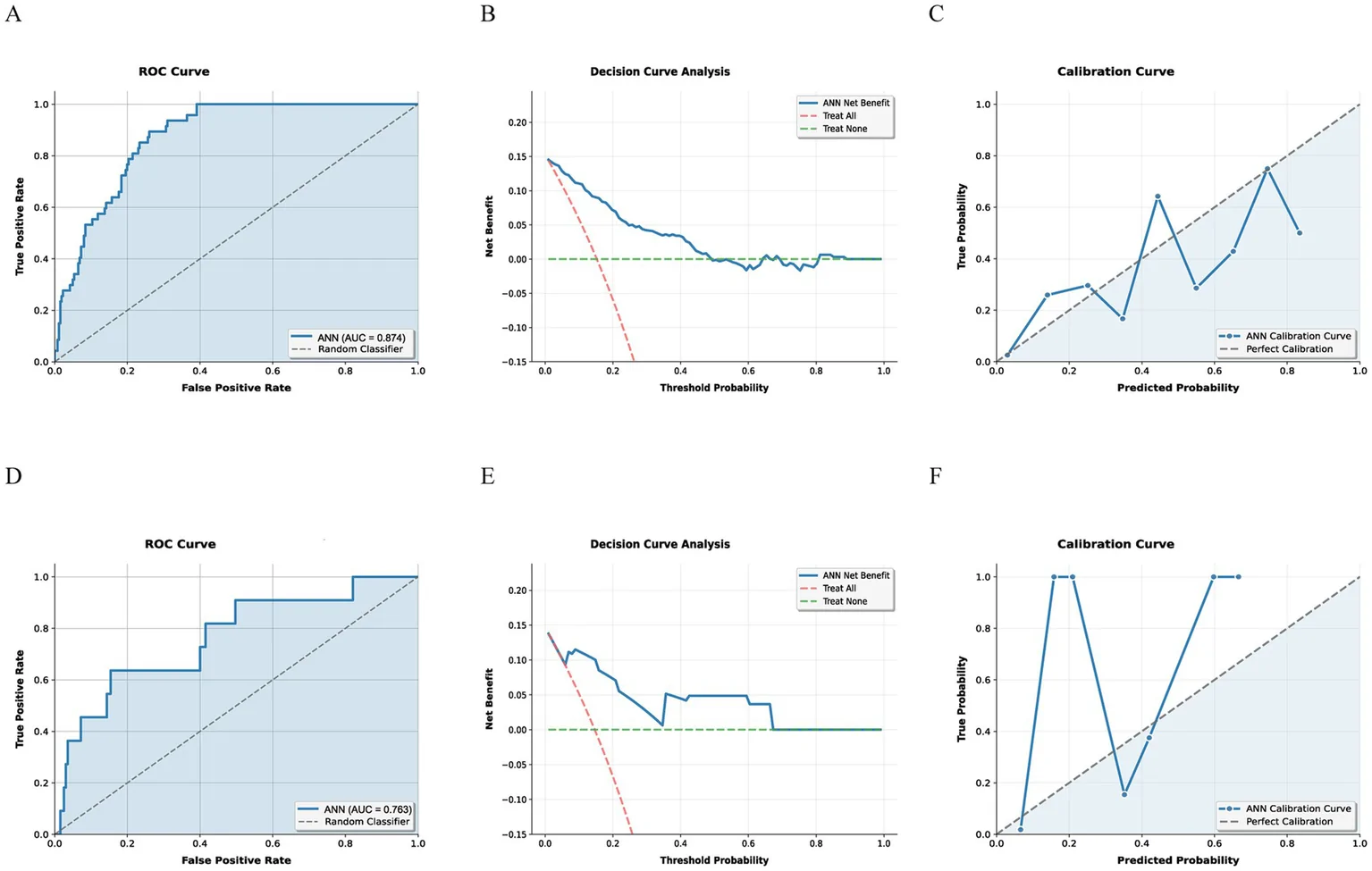
External validation of the ANN model in the eICU-CRD and HIS cohorts. **(A)** ROC curves for eICU-CRD; **(B)** DCA for eICU-CRD; **(C)** Calibration curves for eICU-CRD; **(D)** ROC curves for HIS; **(E)** DCA for HIS; **(F)** Calibration curves for HIS.

### 3.9 SHAP interpretation of the ANN

To interpret the prediction mechanism of the ANN model for 30-day mortality in critically ill patients with NSAH, SHAP analysis was utilized. The SHAP bar plot revealed the magnitude of each feature’s influence on model predictions. The beeswarm plot quantified their directional impact on mortality risk. As shown in Figure 8A, urine output, OASIS, mannitol, vasopressin, and PWR were identified as the top five most important features in descending order of importance. Significantly, PWR ranked among the top features, highlighting its contribution to mortality prediction. Moreover, in Figure 8B, SHAP values > 0 signify a positive impact on mortality risk, while SHAP values < 0 indicate a protective effect. Low PWR values (depicted in blue) strongly increased mortality risk. Conversely, higher PWR (depicted in red) were primarily located in the negative SHAP value range, indicating a potential protective role in short-term risk assessment among critically ill patients with NSAH.

**Figure 8:**
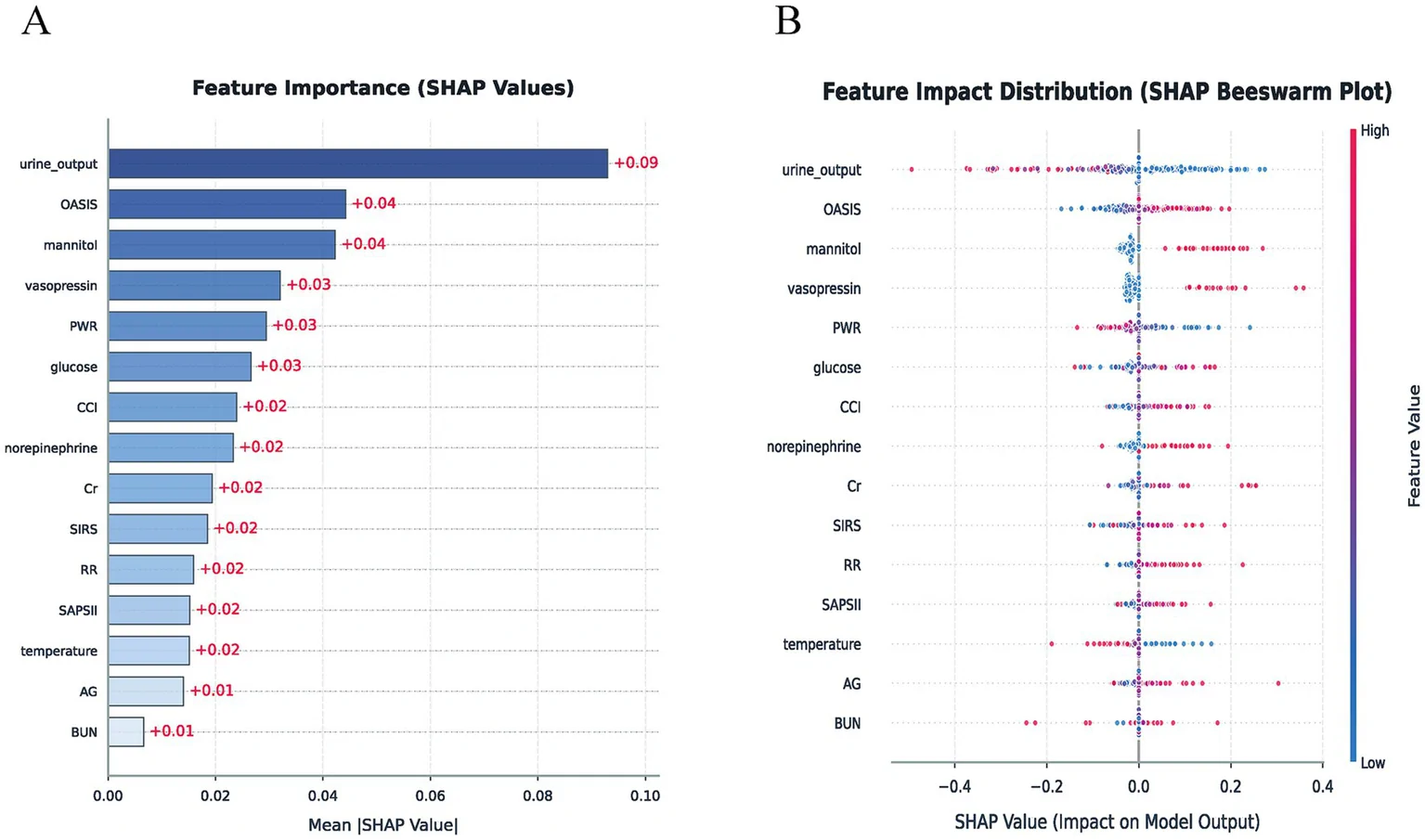
Model explanation using the SHAP method. **(A)** SHAP bar plot; **(B)** SHAP beeswarm plot.

## 4 Discussion

This study was the first systematic investigation into the association between PWR and all-cause mortality in patients with NSAH. The results indicate that reduced PWR levels independently correlate with increased risk of 30-day and 365-day mortality, and this association remained robust even after adjusting for various confounding factors. RCS analysis revealed a linear relationship between PWR and mortality at 30 and 365 days. Additionally, no significant interactions were detected in subgroup analysis.

In the context of the swift advancement of AI, machine learning, a branch of AI, has emerged as a powerful tool for predicting treatment outcomes and patient prognosis in medical research (13). Notably, PWR was identified as a significant predictor in both methods. For the best model, ANN is a powerful machine learning model inspired by biological neural systems, consisting of interacting nodes processing information by weighted connections and nonlinear activation functions. Compared to traditional statistical methods such as LR, ANN demonstrates superior predictive performance, particularly in high-dimensional clinical data (21). To meet clinical practicability, we performed feature selection before modeling. Boruta and LASSO regression algorithms were utilized to ensure rigorous variable screening. In MIMIC-IV, ANN demonstrated the best performance with an AUC of 0.868. To assess the robustness of the ANN model, external validations were conducted utilizing extensive datasets from the eICU-CRD and HIS. The external validation results achieved AUC values of 0.874 and 0.763 for the eICU-CRD and HIS datasets, respectively. The results indicated that the ANN model generalizes well and preserves strong discrimination and calibration across different populations.

PWR has emerged as a novel prognostic marker that integrates information from PLT and WBC, capturing the dynamic balance between inflammatory activity and coagulation status. To date, PWR has shown significant prognostic value in several critical diseases. A large-scale multicenter cohort study by Foy et al. (22) revealed that PWR is more strongly correlated with mortality than other blood count markers and ratios across four acute diseases (COVID-19, acute heart failure, myocardial infarction, and stroke). Cao et al. (9) reported that reduced PWR is independently associated with higher 30-day mortality with atherosclerotic cardiovascular disease. Ko et al. (10) found that PWR may serve as a novel biomarker for predicting the prognosis of pyogenic liver abscesses. Within the realm of neurosurgery, a nomogram that includes PWR accurately predicts long-term survival in intracerebral hemorrhage patients (11). Likewise, low PWR in patients with traumatic acute subdural hematoma is associated with an increased risk of developing post-traumatic acute brain swelling (12). Nevertheless, the clinical value of PWR in prognostic assessment for NSAH remains unclear. Our study was the first to apply PWR to short- and long-term prognostic assessment in critical care for NSAH. Machine learning was also used to develop predictive models. Our findings indicated that integrating PWR into early risk stratification could enhance prognostic evaluation in ICU care for NSAH.

The precise mechanisms linking PWR and mortality in NSAH patients remain unknown. Several potential mechanisms can be suggested in this study. After subarachnoid hemorrhage, blood from ruptured vessels fills the subarachnoid space. The cerebrovascular endothelium suffers damage, exposing collagen and von Willebrand Factor (vWF) and leading to platelet activation via vWF-cleaving metalloprotease (23). Concurrently, thrombin in the bloody cerebrospinal fluid activates platelets through protease-activated receptors-1(PAR-1), accelerating thrombus protein formation and contributing to blood–brain barrier disruption, cerebral edema, neuronal apoptosis, and neuroinflammation (24). This pathological platelet consumption lowers circulating platelet counts. An imbalance of the inflammation–coagulation axis also contributes; it presents as a significant increase in white blood cells accompanied by thrombocytopenia (25, 26). As subarachnoid hemorrhage progresses, complex inflammatory cascades activate, and large numbers of leukocytes, primarily neutrophils, infiltrate and release proinflammatory cytokines (IL-6 and transforming growth factor-β1) and reactive oxygen species (ROS), driving oxidative stress (27). Leukocytes additionally secrete chemokines (CCL22, CXCL12) and platelet-activating factor (PAF), which promote platelet activation (28). Activated platelets also release chemokines (CXCL4 and CCL5) and recruit leukocytes (neutrophils and monocytes) to the endothelium (29). At sites of vascular endothelial injury, leukocytes release neutrophil extracellular traps (NETs). Platelets bind to P-selectin glycoprotein ligand-1 (PSGL-1) on leukocytes through P-selectin on the platelet surface, forming a P-selectin–PSGL-1 complex. This complex promotes platelet and NET aggregation, which triggers immune thrombus formation and amplifies the inflammatory response (30). Low PWR indicates sustained, unregulated inflammation alongside coagulation abnormalities. This disproportion may result in impaired microcirculation, neurological impairment, and multiple organ failure, consequently elevating the mortality hazard among NSAH patients.

Our research presents several advantages: (1) This study utilized extensive real-world datasets, demonstrating that PWR can accurately predict mortality in patients with NSAH. (2) The incidence of missing values for PWR was minimal, which may mitigate selection bias. (3) Compared with prior single-center cohort studies using the MIMIC database, this study analyzed a multinational, multicenter cohort that includes populations from the United States and China, enhancing the reliability and generalizability of the findings. Nonetheless, there are still some limitations. Firstly, longitudinal data on the PWR were lacking, hindering the investigation of its dynamic changes over time. Future research should prioritize examining PWR trajectories. Secondly, despite adjusting for key confounders, unmeasured confounders could influence observed associations, such as the specific location of aneurysm characteristics (location, size, and shape) and grading systems for NSAH (Hunt-Hess score, Fisher score, and WFNS scale). Thirdly, there is a lack of information regarding endovascular coiling. Endovascular coiling represents a critical treatment for NSAH. However, both the MIMIC-IV and eICU-CRD databases contain insufficient documentation of endovascular treatment, resulting in a significant data gap. Fourthly, the retrospective cohort study design inevitably introduces selection bias and cannot establish causality between PWR and mortality in NSAH patients. Additional prospective large-scale studies are necessary to confirm the correlation between PWR and mortality in NSAH patients. Finally, the sensitivity of the ANN model is relatively low (0.453), suggesting that over half of the patients who died were not identified at the existing classification threshold. Consequently, the model should be regarded as a supplementary risk-stratification tool rather than a standalone diagnostic instrument. Future studies that involve larger multicenter cohorts, external validation, and optimization of the classification threshold are necessary to enhance the model’s sensitivity and clinical applicability.

## 5 Conclusion

In summary, our study demonstrated that PWR was an independent predictor of short- and long-term all-cause mortality in patients with NSAH. Lower PWR values were related to higher 30-day and 365-day mortality. The machine learning models developed using PWR and various clinical variables demonstrated robust discrimination and calibration in external multicenter cohorts. The results collectively indicate that PWR could aid in risk stratification and assist clinicians in making clinical decisions for NSAH patients in the ICU. Future multicenter prospective designs are crucial to confirm and expand upon these results.

## Data Availability

The datasets presented in this study can be found in online repositories. The names of the repository/repositories and accession number(s) can be found in the article/Supplementary material.
