# Supplementary material for: Prognostic value of platelet-to-white blood cell ratio in critically ill patients with non-traumatic subarachnoid hemorrhage: a machine learning-driven study of the public database and the hospital information system
Source: Front Neurol. 2026 Sep 30;17:1864288. doi: 10.3389/fneur.2026.1864288 (PMC13661331; doi:10.3389/fneur.2026.1864288)
Supplement: Supplementary file 1 [file Supplementary_file_1.docx]

**Table S1**

Covariables selection

**MIMIC-IV**

**Demographic**

Age

Gender

Race

**Laboratory results**

RBC

WBC

Neutrophils

Lymphocytes

PLT

PWR

Hb

RDW

HCT

PT

APTT

INR

Na+

K+

Cl-

AG

Creatinine

ALB

BUN

AST

ALT

LDH

**Vital signs**

Temperature

RR

SBP

DBP

HR

SpO2

Urine output

**Comorbidities**

Hypertension

Diabetes

Hyperlipidemia

CHF

CPD

Malignant cancer

AKI

Sepsis

CCI

**Clinical severity scores**

APSIII

OASIS

SOFA

GCS

SAPSII

SIRS

**Treatments**

Dobutamine

Dopamine

Epinephrine

Norepinephrine

Vasopressin

Mannitol

Furosemide

Clipping of aneurysm

Ventilator

CRRT

**Clinical outcomes**

30-day mortality

365-day mortality

**Abbreviations:** RBC: red blood cell, WBC: white blood cell, PLT: platelet, PWR: platelet-to-white cell ratio, Hb: Hemoglobin, RDW: red cell distribution width, HCT: hematocrit, PT: Prothrombin Time, APTT: Activated Partial Thromboplastin Time, INR: International Normalized Ratio, Na+: sodium, K+: potassium, Cl-:chloride, AG: anion gap, ALB: albumin, BUN: blood urea nitrogen, AST:aspartate aminotransferase, ALT:alanine aminotransferase, LDH:lactate dehydrogenase, RR: respiratory rate, SBP: systolic blood pressure, DBP: Diastolic blood pressure, HR: heart rate, Spo2: peripheral oxygen saturation, CHF: congestive heart failure, CPD: chronic pulmonary disease, AKI: acute kidney injury, CCI: Charlson Comorbidity Index, APSIII: Acute Physiology Score III, OASIS: Oxford Acute Severity of ILLness Score, SOFA: the Sequential Organ-Failure Assessment, GCS: the Glasgow Coma Scale, SAPSII: Simplified Acute Physiology Score II, SIRS: Systemic Inflammatory Response Syndrome, CRRT: Continuous Renal Replacement Therapy.

| **Table S2**  Variables with missing data | |
| --- | --- |
| Variables | Missing number (%) |
| Age | 0 |
| Gender | 0 |
| Race | 0 |
| RBC | 0 |
| WBC | 0 |
| **Neutrophils** | **746(63%)** |
| **Lymphocytes** | **745(63%)** |
| PLT | 0 |
| Hb | 0 |
| RDW | 0 |
| HCT | 0 |
| PT | 41(3%) |
| APTT | 45(3%) |
| INR | 41(3%) |
| Na+ | 0 |
| K+ | 0 |
| Cl- | 0 |
| AG | 0 |
| Creatinine | 0 |
| **ALB** | **694(59%)** |
| BUN | 0 |
| **AST** | **622(52%)** |
| **ALT** | **622(52%)** |
| **LDH** | **807(68%)** |
| Temperature | 0 |
| RR | 0 |
| SBP | 0 |
| DBP | 0 |
| HR | 0 |
| SpO2 | 0 |
| Urineoutput | 0 |
| Hypertension | 0 |
| Diabetes | 0 |
| Hyperlipidemia | 0 |
| CHF | 0 |
| CPD | 0 |
| Malignant cancer | 0 |
| AKI | 0 |
| Sepsis | 0 |
| CCI | 0 |
| APSIII | 0 |
| OASIS | 0 |
| SOFA | 1 |
| GCS | 1 |
| SAPSII | 0 |
| SIRS | 0 |
| Dobutamine | 0 |
| Dopamine | 0 |
| Epinephrine | 0 |
| Norepinephrine | 0 |
| Vasopressin | 0 |
| Mannitol | 0 |
| Furosemide | 0 |
| Clipping of aneurysm | 0 |
| Ventilator | 0 |
| CRRT | 0 |
| 30-day mortality | 0 |

**Abbreviations:** RBC: red blood cell, WBC: white blood cell, PLT: platelet, Hb: Hemoglobin, RDW: red cell distribution width, HCT: hematocrit, PT: Prothrombin Time, APTT: Activated Partial Thromboplastin Time, INR: International Normalized Ratio, Na+: sodium, K+: potassium, Cl-:chloride, AG: anion gap, ALB: albumin, BUN: blood urea nitrogen, AST: aspartate aminotransferase, ALT: alanine aminotransferase, LDH: lactate dehydrogenase, RR: respiratory rate, SBP: systolic blood pressure, DBP: Diastolic blood pressure, HR: heart rate, Spo2: peripheral oxygen saturation, CHF: congestive heart failure, CPD: chronic pulmonary disease, AKI: acute kidney injury, CCI: Charlson Comorbidity Index, APSIII: Acute Physiology Score III, OASIS: Oxford Acute Severity of ILLness Score, SOFA: the Sequential Organ-Failure Assessment, GCS: the Glasgow Coma Scale, SAPSII: Simplified Acute Physiology Score II, SIRS: Systemic Inflammatory Response Syndrome, CRRT: Continuous Renal Replacement Therapy.

| **Table S3**  Variables with VIF | |
| --- | --- |
| Variables | VIF |
| Age, (years) | 1.71234 |
| Gender, n (%) | 1.285031 |
| Race, n (%) | 1.113174 |
| **RBC, (K/uL)** | **6.515648** |
| WBC, (K/uL) | 3.73316 |
| PLT, (K/uL) | 3.455369 |
| **Hb, (g/L)** | **16.819386** |
| **RDW, (%)** | **16.320542** |
| HCT, (%) | 1.946041 |
| **PT, (s)** | **62.061563** |
| APTT, (s) | 1.141548 |
| **INR** | **61.686011** |
| Na+, (mmol/L) | 2.828565 |
| K+, (mmol/L) | 1.312957 |
| Cl-, (mmol/L) | 3.303404 |
| AG | 1.730283 |
| Creatinine, (mg/dL) | 2.102022 |
| BUN, (mmol/L) | 2.376341 |
| Temperature, (℃) | 1.169068 |
| RR, (times/min) | 1.195913 |
| SBP, (mmHg) | 1.530634 |
| DBP, (mmHg) | 1.46923 |
| HR, (times/min) | 1.534581 |
| SpO2, (%) | 1.15426 |
| Urineoutput, (ml) | 1.617756 |
| Hypertension | 1.191785 |
| Diabetes | 1.473341 |
| Hyperlipidemia | 1.248691 |
| CHF | 1.363261 |
| CPD | 1.073135 |
| Malignant cancer | 1.179747 |
| AKI | 1.224291 |
| Sepsis | 1.840933 |
| CCI | 3.129398 |
| APSIII | 3.951046 |
| OASIS | 3.185976 |
| SOFA | 3.971441 |
| GCS | 1.351659 |
| SAPSII | 4.816918 |
| SIRS | 1.713752 |
| Dobutamine | 1.216357 |
| Dopamine | 1.221599 |
| Variables | VIF |
| Epinephrine | 1.479215 |
| Norepinephrine | 1.941884 |
| Vasopressin | 1.813182 |
| Mannitol | 1.209255 |
| Furosemide | 1.522559 |
| Clipping of aneurysm | 1.070323 |
| Ventilator | 1.349376 |
| CRRT | 1.505708 |

**Abbreviations:** VIF:variance inflation, RBC: red blood cell, WBC: white blood cell, PLT: platelet, Hb: Hemoglobin, RDW: red cell distribution width, HCT: hematocrit, PT: Prothrombin Time, APTT: Activated Partial Thromboplastin Time, INR: International Normalized Ratio, Na+: sodium, K+: potassium, Cl-:chloride, AG: anion gap, BUN: blood urea nitrogen, RR: respiratory rate, SBP: systolic blood pressure, DBP: Diastolic blood pressure, HR: heart rate, Spo2: peripheral oxygen saturation, CHF: congestive heart failure, CPD: chronic pulmonary disease, AKI: acute kidney injury, CCI: Charlson Comorbidity Index, APSIII: Acute Physiology Score III, OASIS: Oxford Acute Severity of ILLness Score, SOFA: the Sequential Organ-Failure Assessment, GCS: the Glasgow Coma Scale, SAPSII: Simplified Acute Physiology Score II, SIRS: Systemic Inflammatory Response Syndrome, CRRT: Continuous Renal Replacement Therapy.

| **Table S4**  Performance evaluation of seven algorithmic models on the internal validation set | | | | | | | | |
| --- | --- | --- | --- | --- | --- | --- | --- | --- |
| Model | AUC | 95% CI Lower | 95% CI Upper | Accuracy | Precision | Sensitivity | F1 score | Specificity |
| LR | 0.852 | 0.814 | 0.889 | 0.819 | 0.622 | 0.373 | 0.467 | 0.939 |
| DT | 0.722 | 0.676 | 0.769 | 0.790 | 0.519 | 0.187 | 0.275 | 0.953 |
| RF | 0.847 | 0.809 | 0.884 | 0.805 | 0.600 | 0.240 | 0.343 | 0.957 |
| LightGBM | 0.847 | 0.809 | 0.884 | 0.799 | 0.538 | 0.373 | 0.441 | 0.914 |
| XGBoost | 0.860 | 0.824 | 0.896 | 0.819 | 0.628 | 0.360 | 0.458 | 0.942 |
| SVM | 0.836 | 0.798 | 0.875 | 0.807 | 0.578 | 0.347 | 0.433 | 0.932 |
| **ANN** | **0.868** | **0.832** | **0.903** | **0.819** | **0.596** | **0.453** | **0.515** | **0.917** |

**Abbreviations:** LR: Logistic Regression, DT: Decision Tree, RF: Random Forest, XGBoost: Extreme Gradient Boosting, LightGBM: Light Gradient Boosting Machine, SVM: Support Vector Machine, ANN: Artificial Neural Network.

**Figure S1**

**(A)** ROC curves on the Training Set; **(B)** DCA on the Training Set; **(C)** Calibration curves on the Training Set.
